# Supplementary material for: Simulating adaptation strategies to offset potential impacts of climate variability and change on maize yields in Embu County, Kenya
Source: PLoS One. 2020 Nov 5;15(11):e0241147. doi: 10.1371/journal.pone.0241147 (PMC7643979; doi:10.1371/journal.pone.0241147)
Supplement: Attachment — Submitted filename: Comments.docx [file pone.0241147.s001.docx]

**General Comments**

This is an interesting article assessing the impacts of climate change on growth and performance of maize using DSSAT and APSIM under RCP4.5/RCP8.5 at the mid and end century. However, the article can be improved by having a reduce and proper structure (section and sub-section) and headings. Some of the section/sub-section was found to be too general and not specific (can be combine with other sub-section) with the research title and objectives. Many sub-section and paragraph was found to be repetitive and redundant. It is preferable to have a sub-section with a heading directly discussing the objectives of the study.

I would strongly advice that a proper step by step procedure on the methodology involved should be prepare to clarify how the objectives was achieved. It was difficult to get a clear picture of the future projection (Tmin, Tmax and Prec) for the study because the authors are employed the 20 GCMs individually. The methodology can be improved by using an ensemble of GCMs, instead of using individual GCMs which introduce a lot of uncertainty in your final output. I would advise the authors to make an ensemble of the 20 GCMs or make an ensemble of the selected GCMs which can better modulate the local climate. Otherwise, the future projection output will have a big uncertainty, confusing and meaningless. The high uncertainty in the output, where some GCMs give positive results, while other GCMs give negative results, would render the results to meaningless as it seems unreliable to at least predict a certain degree of certainty in the future. It is understandable that different GCMs will have its own inherent variability. Therefore, an ensemble methodology should be use to reduce such uncertainty.

It is not clear how the authors get the results for certain output, either through individual GCMs or taking a mean based on 20 GCMs (it should be ensemble, not mean). Due to lack of clarity in the methodology involved in getting the final output of certain section/sub-section, the authors seem to be confuse about what it means by GCM ‘ensemble’.

It would be a major change in the results/output of the research if a proper ensemble method or any method selecting the best GCMs was applied, instead of using 20 individual GCMs (which introduce high uncertainties). I would advise for major revision of the article at its current state because without improve methodology, there will be a high uncertainty in the result/output of this article. Other specific comments can be found below.

**Specific comments**

Abstract:

- Line 9: Website link address can be insert at the Materials and Methods section
- Line 11: It then develops an adaptation strategy that is locally > Then, adaptation strategy was develop that is locally...
- Line 12: that help offset > to offset
- Line 15: but differed in the magnitude of that increase > with different magnitude
- Line 16: higher and those by CanESM2 > higher than CanESM2, INMCM4 and NorESM1-M under both emission scenarios.
- Line 18: abbreviations for minimum temperature (Tmin) and maximum temperature (Tmax) can be use here. Please change for the rest of the manuscript.
- Line 24-27: This sentences is too long to be in the abstract and confusing. What is the meaning of 'short duration varieties' and 'long duration varieties'? Do you mean ‘short duration maize varieties’…?
- Line 34: at national and local levels and make their promotion easier > at national and local level.
- Line 35-38: This sentence is too long. Need to be rephrase and simplify.

Introduction

- Line 57: plant available water? Is this a scientific term?
- Line 107: are viable is required > are viable.
- Line 122: to adapt to the same > for adaptation
- Line 124-126: Developing downscaled location specific climate change scenarios > Downscaling site specific climate change scenarios for various agro-ecological zones (AEZs) of Embu county in Kenya under RCP4.5 and RCP8.5 at the mid and end century period.
- Line 127: on the performance of maize > on maize yields
- Line 128: and to identify key vulnerabilities? What do you mean here? To identify vulnerable area or to identify vulnerability factor?
- Line 129: the objective is for a general agricultural systems or specific to maize agricultural system?

Materials and Methods

Study Location

- Line 157: potential area for what? agriculture? which crop?
- Line 161: do you mean long duration maize varieties?

Crop data and model parameterization

- Line 241: required data > the required data
- Line 248: and the same were derived from the data collected > and the same data was collected...
- Line 249: were derived from the amount > were based on the amount
- Line 265: NO3 and NH4 > NO_3_ and NH_4_
- Line 267: 50 mm and 0.75 m

Results

Observed trends in climate

- Line 278: also in the temperatures > also in the precipitation

Projected changes in future climate

- Line 326: for the crops grown in that AEZ > for the crops grown in the study area.
- Line 341: I think there is a major difference as well between SR and LR season under RCP4.5 although the difference under RCP8.5 is bigger.

Impacts of climate change on maize yields

- Line 389: to mid and end century > at the mid and end century
- Line 403-405: This sentence is redundant. Please remove.
- Line 406-412: It is better to have an ensemble of future projection to reduce the uncertainty rather than using and discussing each individual GCMs which could entail lengthy and contradictory results.
- Line 415-: How did you get the exact percentage result here? Previously, you discuss that there is a range of output by different 20 GCMs being used. You should not take the mean of the output from each GCMs here. Only an ensemble can do.

Effect of management on climate change impacts on maize yields

- Line 429: the effect of variety > the effect of maize variety
- Line 434-: The different and contradictory result might be due to the usage of 20 GCMs individually, instead of using an ensemble of GCMs. This might be the source of high uncertainty in your output by APSIM and DSSAT.
- Line 442: simulation analysis > simulation analysis,
- Line 477: What daoes it means by early planted maize? Can you specify the exact date and duration here?

Impacts under elevated carbon dioxide levels

- Line 493: It is better to have an ensemble of GCMs, rather than individual GCMs result as it will introduce high model uncertainty in your DSSAT and APSIM modelled yields.

Adaptation strategies

- Line 510: performing variety > performing maize variety
- Line 535: which propose intervention do you mean here?

Discussion

- The first paragraph is just a general statement and can be remove or relevantly insert at various part of other section/sub-section.
- Line 552: effectiveness > the effectiveness

Climate variability and change

- This sub-section is too general and doesn't reflect with the research title and doesn't reflect with the research objectives on maize yields. It is better to directly integrate the results being obtained here and relate it with other relevant sub-section which reflect your main findings (related to maize yield).
- The first paragraph is redundant. It is better to directly discuss your results.
- The second paragraph should be discuss in data section
- Line 594: Daily Temperature Range (DTR) > Diurnal Temperature Rang (DTR)
- Line 611: 'we used an ensemble of 20 GCMs' > This statement is confusing with your previous methodology and discussion. Are you using GCMs individually or an ensemble of GCMs? The author should have a proper understanding of what 'ensemble' means.
- Line 612-613: This statement should be discuss in the methods and data section. The reason for selecting only two different emission scenario (RCP4.5 and RCP8.5) and two time period (mid and end century) should be properly justify.
- Line 615-: This section should be in the methods section. It is repetitive.

Impacts of climate variability and change on maize production

- The statement in the first paragraph is general making it lengthy. It should directly discuss the sub-section topic on 'Impacts of climate variability and change on maize production'

Conclusions

- The first paragraph is redundant and repetitive from previous section. It should be remove.
- Line 745: This is the first time that 'multi-model ensemble' being mention, and it was mention at the conclusion. This is confusing. Your results discuss individual GCMs, not an ensemble of GCMs. Please clarify.
- Line 763-: The specific discussion and specific results for the intervention (changing variety, plant density, soil fertility management etc.) are scatter across the article and not properly and directly discuss in sub-section ‘Adaptation strategies’. It is difficult to understand how these interventions play a key role in maize production in relation with climate change impact.
