# Supplementary material for: Simulating adaptation strategies to offset potential impacts of climate variability and change on maize yields in Embu County, Kenya
Source: PLoS One. 2020 Nov 5;15(11):e0241147. doi: 10.1371/journal.pone.0241147 (PMC7643979; doi:10.1371/journal.pone.0241147)
Supplement: Attachment — Submitted filename: Final_Response to comments and suggestions.docx [file pone.0241147.s002.docx]

**Response to comments and suggestions**

To

Shamsuddin Shahid
Academic Editor

PLOS ONE

Dear Editor

We truly appreciate the thorough review and comments offered on our article. After careful consideration of the general and specific comments suggested, we have drafted our responses as shown below and made appropriate changes in the attached revised article

Reviewer #1: (PONE-D-20-14901) Simulating possible Impacts of climate variability and change on Maize production in Embu County, Kenya.

**General comments**

1. This is an interesting article assessing the impacts of climate change on growth and performance of maize using DSSAT and APSIM under RCP4.5/RCP8.5 at the mid and end century. However, the article can be improved by having a reduce and proper structure (section and sub-section) and headings. Some of the section/sub-section was found to be too general and not specific (can be combine with other sub-section) with the research title and objectives. Many sub-section and paragraph was found to be repetitive and redundant. It is preferable to have a sub-section with a heading directly discussing the objectives of the study.

- The structure of the manuscript is revised with additional sub-sections to improve the flow and easy understanding. Redundancy in the manuscript is avoided, authors have made significant changes in the manuscript for the materials, results and discussion sections with introduction of new sub-sections to improve readability of the manuscript.
- Text in the manuscript also updated as follows: Sub-section “Crop data and model parameterization” (2.3) is further divided into sub-sub sections so that readers can understand efforts made by the authors in collecting detailed information for setting up crop simulation model to mimic real conditions. Section (3.6) “Adaptation strategies” is divided into sub-sections highlighting the proposed adaptation options that successfully offset the negative impacts of climate change.

2. I would strongly advice that a proper step by step procedure on the methodology involved should be prepare to clarify how the objectives was achieved. It was difficult to get a clear picture of the future projection (Tmin, Tmax and Prec) for the study because the authors are employed the 20 GCMs individually. The methodology can be improved by using an ensemble of GCMs, instead of using individual GCMs which introduce a lot of uncertainty in your final output. I would advise the authors to make an ensemble of the 20 GCMs or make an ensemble of the selected GCMs which can better modulate the local climate. Otherwise, the future projection output will have a big uncertainty, confusing and meaningless. The high uncertainty in the output, where some GCMs give positive results, while other GCMs give negative results, would render the results to meaningless as it seems unreliable to at least predict a certain degree of certainty in the future. It is understandable that different GCMs will have its own inherent variability. Therefore, an ensemble methodology should be used to reduce such uncertainty.

- We would like to submit that detailed step by step procedure followed which is explained in methods section, further we submit that efforts were made to collect detailed information on individual paraments before setting up the crop-simulations models in the study region. Authors have used 20 GCMs in the study as use of multiple models was suggested to provide more reliable assessment of impacts of climate change on weather sensitive sectors like agriculture (Asseng, 2013; Rosenzweig et al., 2013; Wilby et al., 2009). As suggested in earlier studies several scientists highlighted that cropping systems impact studies of climate change should not be assessed using only one GCM as major source of uncertainty for projections of future climate are from unknown future trajectories of CO_2_ and CH4, emissions, but also due to the highly simplified representation of reality encoded in these models (Wilby et al., 2004). Further the use of multiple models can provide more reliable decision support in climate change impact assessment and assessments of agricultural system vulnerability (Asseng, 2013; Rosenzweig et al., 2013; Wilby et al., 2009). We used 20 available climate models and two emission scenarios (RCP 4.5 and 8.5) in combination with multi-crop models to assess possible potential impacts of climate change on maize production. This study allows researches to understand the full spectrum of impacts of climate change on maize cropping system and further develop robust adaptation options to offset the negative impacts of climate change and at the same time maximize the productivity of maize systems in regions that are benefiting from climate change. Multi-climate model ensemble selection method has its strengths and weaknesses that often prove more trouble than they’re worth in addressing the impacts of climate change particularly on agricultural systems. Hence, we authors were convinced to use 20 GCMs, however in future studies we will try to use GCM ensemble in addition to 20 GCMS to understand its impact on crop yields

3. It is not clear how the authors get the results for certain output, either through individual GCMs or taking a mean based on 20 GCMs (it should be ensemble, not mean). Due to lack of clarity in the methodology involved in getting the final output of certain section/sub-section, the authors seem to be confused about what it means by GCM ‘ensemble’.

- In the present study possible impacts of climate on each AEZs/soil type/maize cultivar/management options deployed are assessed independently. Results presented here are the deviations of maize yields in the future compared to baseline simulations. In order to show the impacts on the maize systems and the factors which are most vulnerable are computed by calculating the mean yield deviations. This is clearly explained in the materials section.

4. would be a major change in the results/output of the research if a proper ensemble method or any method selecting the best GCMs was applied, instead of using 20 individual GCMs (which introduce high uncertainties). I would advise for major revision of the article at its current state because without improve methodology, there will be a high uncertainty in the result/output of this article. Other specific comments can be found below.

- A major revision is performed to improve the manuscript, as mentioned earlier, we have completely revised the manuscript to incorporate the changes suggested by reviewers. After careful evaluation and discussions with global AgMIP leaders on the limitations of multi-climate ensemble for agricultural systems and we have highlighted in the materials and discussion sections why we have used 20 GCMs for the current study. However, we will definitely in future include an ensemble mean of GCMs in all our further studies to understand how the ensemble is impacting the crop yields

**Specific comments**

**Abstract:**

1. Line 9: Website link address can be insert at the Materials and Methods section

- As suggested by the reviewer’s web link is removed for the abstract

2. Line 11: It then develops an adaptation strategy that is locally > Then, adaptation strategy was develop that is locally...

- Changes are incorporated as suggested

3. Line 12: that help offset > to offset

- Changed as suggested

4. Line 15: but differed in the magnitude of that increase > with different magnitude

- Changed as suggested

5. Line 16: higher and those by CanESM2 > higher than CanESM2, INMCM4 and NorESM1-M under both emission scenarios.

- Changed as suggested

6. Line 18: abbreviations for minimum temperature (Tmin) and maximum temperature (Tmax) can be use here. Please change for the rest of the manuscript.

- As suggested Tmin and Tmax are used in the entire manuscript

7. Line 24-27: This sentence is too long to be in the abstract and confusing. What is the meaning of 'short duration varieties' and 'long duration varieties'? Do you mean ‘short duration maize varieties’…?

- Completely re-written, short-duration/ long-duration maize cultivars here refer to the time taken from sowing to crop maturity. In general, short-duration maize cultivars normally take 90 days to mature while, long-duration maize cultivars take 110 to 130 days to complete life-cycle.

8. Line 34: at national and local levels and make their promotion easier > at national and local level.

- Changed as per the suggestion

9. Line 35-38: This sentence is too long. Need to be rephrase and simplify.

- Sentence is completely revised and shorten

**Introduction:**

1. Line 57: plant available water? Is this a scientific term?

- Plant available water (AW) is a technical term: defined as the difference between field capacity, FC, and wilting point, WP. The formula is:

$$AW=FC-WP$$

2. Line 107: are viable is required > are viable.

- Changed as suggested

3. Line 122: to adapt to the same > for adaptation

- Changed as suggested

4. Line 124-126: Developing downscaled location specific climate change scenarios > Downscaling site-specific climate change scenarios for various agro-ecological zones (AEZs) of Embu county in Kenya under RCP4.5 and RCP8.5 at the mid and end century period.

- Changed as suggested

5. Line 127: on the performance of maize > on maize yields

- Changed as suggested

6. Line 128: and to identify key vulnerabilities? What do you mean here? To identify vulnerable area or to identify vulnerability factor?

- Objective is revised as: “Assessing the impacts of climate variability and change on maize yields in different AEZs of Embu county and identify key vulnerabilities to climate factors”.

7. Line 129: the objective is for a general agricultural system or specific to maize agricultural system?

- The objective is particularly focused for maize cropping system in eastern Kenya; however, this methodology remains same for other crops with different adaptation options

**Materials and Methods:**

**Study Location:**

1. Line 157: potential area for what? agriculture? which crop?

- Low/High potential here refers to agricultural crops, sentence is revised to represent maize system in this context.

2. Line 161: do you mean long duration maize varieties?

- In general, short-duration maize cultivars normally take 90 days to mature while, long-duration maize cultivars take 110 to 130 days to complete life-cycle. In the entire manuscript maize varieties are replaced with maize cultivars to avoid any confusion.

**Crop data and model parameterization:**

1. Line 241: required data > the required data

- Changed as suggested

2. Line 248: and the same were derived from the data collected > and the same data was collected...

- Changed as suggested

3. Line 249: were derived from the amount > were based on the amount

- Changed as suggested

4. Line 265: NO3 and NH4 > NO_3_ and NH_4_

- Changed as suggested, care has taken to represent the subscript for chemical formulas in the entire manuscript

5. Line 267: 50 mm and 0.75 m

- The sentence is revised and both depth and plant spacing are represent with same unit (cm)

**Results:**

**Observed trends in climate:**

1. Line 278: also in the temperatures > also in the precipitation

- Revised as suggested

**Projected changes in future climate:**

2. Line 326: for the crops grown in that AEZ > for the crops grown in the study area.

- Revised as suggested

3. Line 341: I think there is a major difference as well between SR and LR season under RCP4.5 although the difference under RCP8.5 is bigger.

- Revised as suggested

**Impacts of climate change on maize yields:**

1. Line 389: to mid and end century > at the mid and end century

- Revised as suggested

2. Line 403-405: This sentence is redundant. Please remove.

- Removed as suggested

3. Line 406-412: It is better to have an ensemble of future projection to reduce the uncertainty rather than using and discussing each individual GCMs which could entail lengthy and contradictory results.

- Several researchers have made a case for seeking consensus amongst using multi-climate ensemble and individual GCMs projections. Agricultural systems responses to climate change is not linear to projected changes in surface temperatures or rainfall, it is a complex interaction where climate along with cultivar, soil and crop management options have significant impact on growth, development and finally productivity. In this study we used multi-climate models (20 GCMs) to assess the possible impacts of climate change to cover broad spectrum of impacts so that location specific adaptation options can be developed to cover wide range of impacts.

4. Line 415-: How did you get the exact percentage result here? Previously, you discuss that there is a range of output by different 20 GCMs being used. You should not take the mean of the output from each GCMs here. Only an ensemble can do.

- Crop model simulated future maize yields deviation from baseline (seasons independently) are computed for each GCM. The average deviation is reported here. In the revised manuscript the range of deviation along with mean deviation are provided to avoid any confusion. Studies using multi-climate models particularly, agricultural systems the impact is often represent as mean deviation from baseline unlike meteorological studies where projections are characterized using multi-climate model ensembles. Few studies used multi-climate model ensembles, however, they failed to represent the full range of possible impacts of climate change on agricultural systems and the strategic adaptation options developed failed to cover all the possible climate projections.

**Effect of management on climate change impacts on maize yields:**

1. Line 429: the effect of variety > the effect of maize variety

- Revised as suggested

2. Line 434-: The different and contradictory result might be due to the usage of 20 GCMs individually, instead of using an ensemble of GCMs. This might be the source of high uncertainty in your output by APSIM and DSSAT.

- Crop simulations models APSIM and DSSAT have structural differences in simulation function. Large variation among the crop models because of different assumptions for parameter functions [Boote et al., 2013] like cardinal temperature (CR), photoperiod and low temperature sensitivity. As a result, both the crop simulation models marginally differed in assessing the impacts of climate change on maize cultivars.

3. Line 442: simulation analysis > simulation analysis,

- Revised as suggested

4. Line 477: What does it means by early planted maize? Can you specify the exact date and duration here?

- Early planting maize here refers to early sowing relative to normal sowing date. Since the planting window changes for each season and AEZs the details are not provided in the text. However, the changes in planting dates (early planting) for each season and AEZ are provided in table 7 and which is referred in the text.

Impacts under elevated carbon dioxide levels

1. Line 493: It is better to have an ensemble of GCMs, rather than individual GCMs result as it will introduce high model uncertainty in your DSSAT and APSIM modelled yields.

- As explained earlier, the study assesses the combined effects of climate change, soil, maize cultivar, AEZ, CO_2_ fertilization and management options on future maize systems to cover full range of possible impacts. This allows policy makes and stakeholders to understand wide range of impacts under climate scenario and there by develop adaptations that considers all possible impacts on maize systems.

**Adaptation strategies:**

1. Line 510: performing variety > performing maize variety

- Revised as suggested and variety is replaced with cultivar in the entire manuscript

2. Line 535: which propose intervention do you mean here?

- Here “proposed interventions” refers to the adaption strategies such as changing planting dates, plant population, fertilizer amounts and maize cultivars. The whole section is revised and sub-sections are introduced to highlight the adaptation strategies that were recommended in this manuscript for easy understanding to readers.

**Discussion:**

1. The first paragraph is just a general statement and can be remove or relevantly insert at various part of other section/sub-section.

- Whole discussing is revised and we would like to thanks the reviewers for the critical comments that helped in improving the manuscript. As suggested the first paragraph is removed

2. Line 552: effectiveness > the effectiveness

- Changes incorporated as suggested

Climate variability and change

3. This sub-section is too general and doesn't reflect with the research title and doesn't reflect with the research objectives on maize yields. It is better to directly integrate the results being obtained here and relate it with other relevant sub-section which reflect your main findings (related to maize yield).

- The section is completely revised as per the suggestions

4. The first paragraph is redundant. It is better to directly discuss your results.

- Revised as per the suggestions and removed repeated text

5. The second paragraph should be discuss in data section

- Revised and moved as per the suggestion

6. Line 594: Daily Temperature Range (DTR) > Diurnal Temperature Rang (DTR)

- Revised and changed Daily to Diurnal

7. Line 611: 'we used an ensemble of 20 GCMs' > This statement is confusing with your previous methodology and discussion. Are you using GCMs individually or an ensemble of GCMs? The author should have a proper understanding of what 'ensemble' means.

- Revised the complete section to avoid the confusion on ensemble, in this study multi-climate model ensemble is not used rather 20 GCMs were used that are able to represent broadly from the distribution of projections.

8. Line 612-613: This statement should be discuss in the methods and data section. The reason for selecting only two different emission scenario (RCP4.5 and RCP8.5) and two time period (mid and end century) should be properly justify.

- As per the suggestion this is moved to material section and justified why the study uses RCP 4.5 and 8.5.

9. Line 615-: This section should be in the methods section. It is repetitive.

- Repetitive text in the manuscript are removed and as per the suggestions from reviewers it is moved to methods section

Impacts of climate variability and change on maize production

1. The statement in the first paragraph is general making it lengthy. It should directly discuss the sub-section topic on 'Impacts of climate variability and change on maize production'

- Revised and general text is removed as per the suggestion

**Conclusions:**

1. The first paragraph is redundant and repetitive from previous section. It should be remove.

- Removed and the whole section is revised

2. Line 745: This is the first time that 'multi-model ensemble' being mention, and it was mention at the conclusion. This is confusing. Your results discuss individual GCMs, not an ensemble of GCMs. Please clarify.

- Revised the whole sections and removed multi-climate ensemble to avoid any confusion to readers

3. Line 763-: The specific discussion and specific results for the intervention (changing variety, plant density, soil fertility management etc.) are scatter across the article and not properly and directly discuss in sub-section ‘Adaptation strategies’. It is difficult to understand how these interventions play a key role in maize production in relation with climate change impact.

- In the discussion section particularly, ‘Adaptation strategies’ is completely revised and sub-sections were included to describe how the proposed interventions influenced to offset the negative impacts of climate change and at the same time in regions where the projected impacts are beneficial to maize systems, adaptation options maximized maize yields even in low input systems

Reviewer #2: (PONE-D-20-14901) Simulating possible Impacts of climate variability and change on Maize production in Embu County, Kenya.

Abstract:

1. L9: Eliminate “([www.agmip.org)](http://www.agmip.org))”.

- Removed web link as suggested

2. There are mixed verb tenses, somewhere use the present tense “L8: The study assesses…..”, and other places use the past tense, “L12: The study used…”. Please use the present or past tense in the whole of the paper.

- Carefully revised the entire manuscript to avoid mixed verb tenses

3. Please rewrite lines 13 to 15.

- Revised the lines between 13 to 15

4. It would be better to highlight your contribution, more clearly in abstract.

- Revised abstract to highlight our recommended adaptions we proposed

Introduction:

1. The authors should highlight the research significant, more clearly in the introduction section.

- Revised introduction as suggested to highlight significance of the current research

2. There are some grammatical errors in this section. Please check whole the section carefully.

- Authors and AgMIP leaders improved the manuscript including grammatical errors

3. References are not always relevant and up-to-date. This reviewer suggests the inclusion of a few relevant publications. The list is given below:

- <https://rmets.onlinelibrary.wiley.com/doi/abs/10.1002/joc.6307>
- <https://link.springer.com/article/10.1007/s11368-020-02632->0
- <https://link.springer.com/article/10.1007/s00704-019-02979->6
- <https://www.sciencedirect.com/science/article/pii/S0168192319302199>
- References provided are used where ever they are applicable

Method:

1. It is mentioned in L133 that the Embu County in Kenya is adopted as the case study. What are other feasible alternatives? What are the advantages of adopting this particular case study over others in this case? How will this affect the results? The authors should provide more details on this

- The study selected Embu county for the following:
- Embu county is one of the potential agricultural regions characterised by a range of agro-ecological zones ranging from highlands to lowlands.
- The county was selected based on its representativeness of the country’s major agro-ecological zones and based on the availability of the data (crop, soil and climate) required to parameterize the crop simulation models.
- The details are provided in the section 2.1 “Study location”

2. It is mentioned in L181 that historical records of 1980 to 2010 are taken. Why are more recent data not included in the study? Is there any difficulty in obtaining more recent data? Are there any changes to the situation in recent years? What are its effects on the result?

- Efforts were made to collect latest data, however, due to limitations in sharing climate records as per the country policy with CGIAR institutes this study only considered to represent baseline between 1980-2010

Results and discussion

1. From Figure 6, it is evident that the authors obtained poor performance (R2: 0.33-0.44) during the validation phase. Is it a reliable model for crop simulating?

- The poor performance of the crop simulation models in reproducing the maize yields for the year 2013 are due to large number of factors which are discussed in detail in the result section (3.3 Crop model calibration and validation). Crop simulation model’s performance in reproducing experiment yields are in good agreement while in the farmers fields the poor correlation is due to number factors such as differences in interpreting and translating farmer description of the resource endowment into model parameters, inability of the models to capture the effects of biotic stresses such as pests, diseases and weeds, inaccuracies in estimating per hectare yields from bags per plot as reported by farmers and inaccuracies in defining the initial conditions. However, the simulated long-term yields of different AEZs reflected the trends in the yields reported by farmers fairly well, especially in the low potential LM4 and LM5 AEZs. In these AEZs, high moisture stress is the major yield limiting factor and this to a large extent masks the relatively low effect of other management practices and also the influence of differences in the resource base.

Conclusion:

1. In the conclusion section, the limitations of this study suggested improvements in this work, and future directions should be highlighted.

- The limitations of the study and further improvements required are described in the conclusions as suggested
