# Supplementary material for: Simulating adaptation strategies to offset potential impacts of climate variability and change on maize yields in Embu County, Kenya
Source: PLoS One. 2020 Nov 5;15(11):e0241147. doi: 10.1371/journal.pone.0241147 (PMC7643979; doi:10.1371/journal.pone.0241147)
Supplement: Attachment — Submitted filename: Comments.docx [file pone.0241147.s003.docx]

**General Comments**

The manuscript has been greatly improved by the authors after the first revision. However, there is still few improvements on the arrangement of the section/sub-section that can be done (refer to the specific comments). In term of methodology, although the authors have reply properly the comments given, some of it wasn’t inserted into the manuscript (refer to the specific comments). The methods and results are still mixed-up in some of the section/sub-section which the authors can make in a more proper order and sequence. Some clarification on how the future changes was calculated has been reply through peer-review comments but wasn’t mentioned anywhere in the manuscript. There are some minor but substantial details on the results and discussion that need to be revised.

Other specific comments can be found below.

**Specific comments**

Abstract:

- Line 19: There are 20 GCMs used. Therefore, projection by HadGEM2-CC, HadGEM2-ES, and MIROC-ESM should be higher than the rest of 17 GCMs. Why is it mention here, projection of HadGEM2-CC, HadGEM2-ES, and MIROC-ESM is higher than CanESM2, INMCM4 and NorESM1-M only?
- Line 31: Are you 'develop' the adaptation practices or 'incorporate' the adaptation practices into the model?
- Line 33: I think you are incorporating the operational adaptation strategies using the readily available technologies here, not develop the operational adaptation strategies using the readily available technologies.

Section 1. Introduction

- Line 63: which are > which have a marginal
- Line 63: adding to this > adding to this,
- Line 125: Instead of using the term climate, a more specific term, rainfall, Tmin, Tmax (climate indices that you assess) should be mention here. Climate can be a lot of things.
- Line 127: Repeated abbreviation for AEZs
- Line 127: various AEZs, namely,... > the zones used in the study should be mentioned here.
- Line 130: Are you developing adaptation options?

Section 2.1 Study Location

- Line 141: rising > up to
- Line 157: AEZs > AEZs of
- Line 163: higher use > higher application

2.2 Assessing current climate variability and future climate conditions

- Line 166: Assessing? Are you making any assessment here? There is no discussion on the assessment of current climate variability in this sub-section. The heading is confusing.
- Line 181: Please be notify that the font size is different here. Please check throughout the manuscript.
- Line 190: pathway are > pathway
- Line 193-196: The equation number for equation (1)-(4) should be align right and the equation should be centre.

Section 2.3

- Line 234: I don't think you need to give this heading. Sub-heading 2.31 can be change to sub-heading 2.3

2.3.1 Crop Simulation Models (CSMs)

- Line 241: were used to assess > to assess
- Line 246: Please recheck if there is any more repetitive abbreviation throughout the manuscript.

2.3.2 Model parameterization

- 255: Sub-heading 2.3.2 can be change to 2.4. While sub-section Farm and farm management data can be change to 2.4.1 etc for the rest of sub-heading.

Farm and farm management data:

- Line 271-276: Please re-phrase this sentence.
- Line 281: During In the survey > During the survey
- Line 283: This formed?

Model calibration and validation:

- Line 312-323: This discussion should be put into results or discussion section.

Crop simulations:

- Line 332: NO3, NH4 > NO_3_, NH_4_
- Line 337: and or > and
- Line 342: crop simulation model > CSM

3. Results

- There should be a sub-section under Results to discuss the performance of APSIM and DSSAT in term of calibration and validation of the model.

3.1 Trends in observed climatic conditions

- Line 359: How do you measure the trend? Simple Linear Regression?
- Line 360: delete 'and in the rate of increase'
- Line 361: Why are you comparing Tmax and Tmin for period 2001-2010 with period 1981-1990? You can make a simple regression line to see the increasing rate of Tmin/Tmax from the whole historical period of 1981-2010.
- Line 367: If you are using simple linear regression for trend, you can give the increasing/decreasing rate here.
- Line 373-378: You discuss the results in the previous paragraph. Only now, you discuss the method that you use. This is not in proper order. Also, please give briefly the method that you use for trend analysis in the method section. Not in results section.

3.2 Projected changes in climate conditions

- Line 380: surface temperature > use directly the specific temperature indice that you employ, Tmin and Tmax. Surface temperature can be a lot of things.
- Line 385: You are employing 20 GCMs, but these 4 GCMs (HadGEM2-CC, HadGEM2-ES, IPSL-CM5A-MR, IPSL-CM5A-LR) is higher compare to only other 4 GCMs (CanESM2, INMCM4, MRI-CGCM3, and NorESM1-M)? This is confusing.
- Line 388: Projected increase was being compare with which historical period?
- Line 391: Please use a consistent decimal places for temperature throughout the manuscript.
- Line 397: 3.5 ^o^C > 3.5^o^C, make similar correction throughout the manuscript
- Line 398: Give numerical value for the result and discussion. What is the value for current temperature? What is value for low temperature event? What is value for high temperature event?
- Line 400: sift > shift

Impacts across AEZs:

- This can be sub-heading 3.2.1. Do the same for the rest.

Impacts across seasons:

- Line 447: delete 'DSSAT'
- Line 451: what is the numerical value for baseline yields?

Impacts of plant population:

- Line 496: population > plant population

Impacts of soil fertility and nitrogen application:

- Line 502: How exactly climate change give positive impacts? Increase rainfall/temperature?

3.3 Adaptation strategies

- Line 527: What exactly is adapted technology that you mentioned here?
- Line 531: All of this results discussing about adapted technology? or certain management practice? Please make it clear. In the first paragraph of this section, you are talking about various management practice, then you are talking about adapted technology. Now, the results are talking about which one? This is confusing.

4.1 Assessing variability in the current and projected climatic conditions

- Section 4.1 > This heading is unnecessary

4.1.1 Climate Variability

- Line 567: variability in term of temporal or spatial?

4.2 Impacts of climate variability and change on maize production

- Line 644: was done carried out > was carried out
- Line 659: AEZs > AEZs of
- Line 660: AEZs > AEZs of

4.3 Adapting to climate change

- Line 705: This sub-section seems to be a general statement. May be can briefly incorporate in the conclusion.

Figure 2

- The outside borderline box should be remove. Do the same for all figure except fIg. 1 as the borderline box is require for lat/lon information.

Figure 5

- The Legend can be improve
- Please re-check the font size of the caption

Figure 7

- Please put dot line between AEZs. The series overlap can be made to 0%. Do the same for Fig. 9 as well.

Figure 8

- Series overlap can be made to 0% for each GCM

Table 1

- Table can be improved. No color shading should be use and a proper bordering line should be employ. Please refer to other research article on how to prepare a proper table for research publication. Do the same for all Table.
