# Supplementary material for: Simulating adaptation strategies to offset potential impacts of climate variability and change on maize yields in Embu County, Kenya
Source: PLoS One. 2020 Nov 5;15(11):e0241147. doi: 10.1371/journal.pone.0241147 (PMC7643979; doi:10.1371/journal.pone.0241147)
Supplement: Attachment — Submitted filename: Response to comments and suggestions19Sept.docx [file pone.0241147.s004.docx]

**Response to comments and suggestions**

To

Shamsuddin Shahid
Academic Editor

PLOS ONE

Dear Editor

We truly appreciate the thorough review and comments offered on our article. After careful consideration of the general and specific comments suggested, we have drafted our responses as shown below and made appropriate changes in the attached revised article

Reviewer #1: (PONE-D-20-14901) Simulating possible Impacts of climate variability and change on Maize production in Embu County, Kenya.

**General Comments**

The manuscript has been greatly improved by the authors after the first revision. However, there is still few improvements on the arrangement of the section/sub-section that can be done (refer to the specific comments). In term of methodology, although the authors have reply properly the comments given, some of it wasn’t inserted into the manuscript (refer to the specific comments). The methods and results are still mixed-up in some of the section/sub-section which the authors can make in a more proper order and sequence. Some clarification on how the future changes was calculated has been reply through peer-review comments but wasn’t mentioned anywhere in the manuscript. There are some minor but substantial details on the results and discussion that need to be revised.

Other specific comments can be found below.

- we author’s thanks the reviewers for their positive comments for the improvements made on our manuscript
- The structure of the manuscript is revised as per the suggestions of the reviewer, particularly on the rearrangement of section/sub-sections. Authors have made changes in the manuscript for the for all the sections as suggested.
- Text in the manuscript also updated as follows: details of the non-parametric Mann Kendall test is incorporated in the materials and methods section (under 2.2). As suggested a new section on Crop Simulation Models (CSMs) is changes to sub-section (2.3)

**Specific comments**

Abstract:

- Line 19: There are 20 GCMs used. Therefore, projection by HadGEM2-CC, HadGEM2-ES, and MIROC-ESM should be higher than the rest of 17 GCMs. Why is it mention here, projection of HadGEM2-CC, HadGEM2-ES, and MIROC-ESM is higher than CanESM2, INMCM4 and NorESM1-M only?
- Line 19 is changed to “Projections by HadGEM2-CC, HadGEM2-ES, and MIROC-ESM tend to be higher than rest of 17 CMIP5 climate models under both emission scenarios.” Here earlier we compare the results with the top 6 projected changes in the selected CMIP5 GCMs.
- Line 31: Are you 'develop' the adaptation practices or 'incorporate' the adaptation practices into the model?
- Development of adaptation practices here is a strategic approach in identifying the best performing practices. As per the suggestion of the reviewer we have replaced development of adaptation practices as identified practices. “Using the differential impacts of climate change, a strategy to adapt maize cultivation to climate change in all the five AEZs was identified by consolidating those practices that contributed to increased yields under climate change.”
- Line 33: I think you are incorporating the operational adaptation strategies using the readily available technologies here, not develop the operational adaptation strategies using the readily available technologies.
- As responded earlier, we (authors) have replaced “development” with “identified”, across the manuscript.

**Section 1. Introduction**

- Line 63: which are > which have a marginal
- Changed as per the suggestion
- Line 63: adding to this > adding to this,
- Changed as per the suggestion
- Line 125: Instead of using the term climate, a more specific term, rainfall, Tmin, Tmax (climate indices that you assess) should be mention here. Climate can be a lot of things.
- As recommended, the term climate is replaced with specific climate variable such as rainfall, Tmax and Tmin
- Line 127: Repeated abbreviation for AEZs
- Across the manuscript repeated abbreviation for agro-ecological zines are replaced with AEZs
- Line 127: various AEZs, namely,... > the zones used in the study should be mentioned here.
- As suggested by the reviewer, the names of the AEZs are mentioned in the objectives and details of the AEZs are further provided in the materials and methods section under section 2.1 study location
- Line 130: Are you developing adaptation options?
- Development of adaptation options in this context is a strategy for identifying best performing crop management practices. However, as responded earlier we have replaced “development” with “identified” across the manuscript.

**Section 2.1 Study Location**

- Line 141: rising > up to
- Changed as suggested
- Line 157: AEZs > AEZs of
- Changed as suggested
- Line 163: higher use > higher application
- Changed as suggested

**2.2 Assessing current climate variability and future climate conditions**

- Line 166: Assessing? Are you making any assessment here? There is no discussion on the assessment of current climate variability in this sub-section. The heading is confusing.
- As suggested by the reviewer, assessment is removed from the sub heading and replaced with “Current climate variability and future climate conditions”
- Line 181: Please be notify that the font size is different here. Please check throughout the manuscript.
- A through check is performed across the manuscript to maintain same font size and type.
- Line 190: pathway are > pathway
- Changed as suggested
- Line 193-196: The equation number for equation (1)-(4) should be align right and the equation should be centre.
- Changed as suggested, equitation’s number are aligned right and equitation is centred

**Section 2.3**

- Line 234: I don't think you need to give this heading. Sub-heading 2.31 can be change to sub-heading 2.3
- Changed as suggested

**2.3.1 Crop Simulation Models (CSMs)**

- Line 241: were used to assess > to assess
- Changed as suggested
- Line 246: Please recheck if there is any more repetitive abbreviation throughout the manuscript.
- A through check is performed across the manuscript to replace the repetitive abbreviation

**2.3.2 Model parameterization**

- 255: Sub-heading 2.3.2 can be change to 2.4. While sub-section Farm and farm management data can be change to 2.4.1 etc for the rest of sub-heading.
- Changed as suggested

**Farm and farm management data:**

- Line 271-276: Please re-phrase this sentence.
- The sentences are re-phrased in the section 2.4.2 Soil Data
- Line 281: During In the survey > During the survey
- Changed as suggested
- Line 283: This formed?
- Removed and re-phrased

**Model calibration and validation:**

- Line 312-323: This discussion should be put into results or discussion section.
- Calibration and validation approach only are presented in the methods section and as suggested we have the results of this section to 3.2 Projected changes in climate conditions

**Crop simulations:**

- Line 332: NO3, NH4 > NO_3_, NH_4_
- Changed as suggested
- Line 337: and or > and
- Changed as suggested
- Line 342: crop simulation model > CSM
- Changed as suggested

**3. Results**

- There should be a sub-section under Results to discuss the performance of APSIM and DSSAT in term of calibration and validation of the model.
- As suggested by the reviewer, a new section on crop model’s calibration and validation is incorporated in the results section. (3.2.1 Calibration and Validation of APSIM and DSSAT)

**3.1 Trends in observed climatic conditions**

- Line 359: How do you measure the trend? Simple Linear Regression?
- Mann Kendall Tau’s test is used to measure the significance of trends, as suggested by the reviewer, we have provided the details of Mann Kendall test in the materials and methods section (under 2.2)
- Line 360: delete 'and in the rate of increase'
- Changed as suggested
- Line 361: Why are you comparing Tmax and Tmin for period 2001-2010 with period 1981-1990? You can make a simple regression line to see the increasing rate of Tmin/Tmax from the whole historical period of 1981-2010.
- Here we are comparing temperatures between two time periods, 1981-1990 and 2001-2010, to show the changes that have occurred in the historical time series. This approach enables agricultural researches to understand the changes in growing degrees days to complete each phenological stage and furthers allows to identify suitable maize cultivar with the changing climate.
- Line 367: If you are using simple linear regression for trend, you can give the increasing/decreasing rate here.
- There is no significant trend in observed rainfall amounts in both seasonal and annual rainfall and across the locations. However, the inter-annual variability in is very high particularly during the SR season. To capture the variability, a ten-year moving Coefficient of Variation (CV) of SR season rainfall is computed which displayed an increase in variability by 30 % in 80’s and substantially increased up to 50% during 2001-2010 period. Here we used different decadal statistics to show the changes in variability which play a key role in changes in crop management practices. This helps the agricultural researchers to understand the associated changes in climate variables (rainfall and temperatures) during each decade and thus, strategic crop adaptation measures can be identified to build resilience to the changing environments.
- Line 373-378: You discuss the results in the previous paragraph. Only now, you discuss the method that you use. This is not in proper order. Also, please give briefly the method that you use for trend analysis in the method section. Not in results section.
- As suggested by the reviewer, we have provided the details of Mann Kendall Tau test in the materials and methods section and the section is revised.

**3.2 Projected changes in climate conditions**

- Line 380: surface temperature > use directly the specific temperature indices that you employ, Tmin and Tmax. Surface temperature can be a lot of things.
- Replaced “Surface Temperatures” with Tmax and Tmin as suggested by the reviewer
- Line 385: You are employing 20 GCMs, but these 4 GCMs (HadGEM2-CC, HadGEM2-ES, IPSL-CM5A-MR, IPSL-CM5A-LR) is higher compare to only other 4 GCMs (CanESM2, INMCM4, MRI-CGCM3, and NorESM1-M)? This is confusing.
- Line 385 is changed to “In general, Tmin and Tmax projections by HadGEM2-CC, HadGEM2-ES, IPSL-CM5A-MR, IPSL-CM5A-LR and MIROC-ESM were found to be higher compared to 17 CMIP5 GCMs selected for the current study.” Here earlier we compare the results with the top 6 projected changes projected by the selected CMIP5 GCMs.
- Line 388: Projected increase was being compare with which historical period?
- The projected changes were compared with the baseline data used in the current study that is from 1980 to 2010.
- Line 391: Please use a consistent decimal place for temperature throughout the manuscript.
- Throughout the manuscript authors revised so that consistency is maintained for temperatures.
- Line 397: 3.5 ^o^C > 3.5^o^C, make similar correction throughout the manuscript
- Throughout the manuscript authors revised to maintain consistency
- Line 398: Give numerical value for the result and discussion. What is the value for current temperature? What is value for low temperature event? What is value for high temperature event?
- Based on the Probability Density Function (PDF) analysis, across the four locations the low temperature event is 9°C (for the baseline Tmin) and high temperature event (above 34°C, Tmax for baseline climate), which is reported in the manuscript now and the same is displayed in Figure 6)
- Line 400: sift > shift
- Changed as suggested

**Impacts across AEZs:**

- This can be sub-heading 3.2.1. Do the same for the rest.
- As suggested the sub-heading is revised

**Impacts across seasons:**

- Line 447: delete 'DSSAT'
- Changed as suggested
- Line 451: what is the numerical value for baseline yields?
- Average maize yields across the surveyed farms for SR season is 1.5 t/ha and LR season is 1.4t/ha. For Embu station SR= 2.3 t/ha, LR= 1.8 t/ha; Ishiara station: SR=1.1 t/ha, LR= 1.2 t/ha; Karurumo: SR= 1.6 t/ha, LR= 1.5 t/ha and Kindaruma: SR= 0.8 t/ha, LR= 0.9 t/ha

**Impacts of plant population:**

- Line 496: population > plant population
- Changed as suggested

**Impacts of soil fertility and nitrogen application:**

- Line 502: How exactly climate change gives positive impacts? Increase rainfall/temperature?
- Increase in maize yields in the future are due to increased rainfall temperatures and elevated CO_2_ levels in the atmosphere. As discussed in the discussion section, even though the temperature is increasing in the future they are still within the crop optimal range. In the baseline simulations CSMs experienced low temperature stress that is average daily temperature less than 10°C. In CSMs cardinal temperature are as follows: (minimum temperature: 10°C, optimum temperature: 26 to 31.1 °C maximum temperature: 38 °C).

**3.3 Adaptation strategies**

- Line 527: What exactly is adapted technology that you mentioned here?
- Here adapted technology refers to a set of management practices that included best performing maize cultivar, planting time, plant population and fertilizer amount (Table 7).
- Line 531: All of this result discussing about adapted technology? or certain management practice? Please make it clear. In the first paragraph of this section, you are talking about various management practice, then you are talking about adapted technology. Now, the results are talking about which one? This is confusing.
- Adaptation technology as referred above is a set of management practices that are able to offset negative impacts of climate change on agricultural crops. In general, and in particularly in crop modelling studies the set of crop management practices that are identified to offset the negative impacts of climate change are referred as adaption measures/technology. However, we have revised the “adapted technology” as “adapted crop management practices” to avoid any confusion.

**4.1 Assessing variability in the current and projected climatic conditions**

- Section 4.1 > This heading is unnecessary
- As suggested by the reviewer, the sub-heading is removed

4.1.1 Climate Variability

- Line 567: variability in term of temporal or spatial?
- Temporal variability in rainfall as discussed in the results section 3.1	Trends in observed climatic conditions.

**4.2 Impacts of climate variability and change on maize production**

- Line 644: was done carried out > was carried out
- Changed as suggested
- Line 659: AEZs > AEZs of
- Changed as suggested
- Line 660: AEZs > AEZs of
- Changed as suggested

**4.3 Adapting to climate change**

- Line 705: This sub-section seems to be a general statement. May be can briefly incorporate in the conclusion.
- As suggested by the reviewer we removed the subsection “Adapting to climate change” and some important message from the this section were briefly incorporated in conclusion section

**Figure 2**

- The outside borderline box should be remove. Do the same for all figure except fIg. 1 as the borderline box is require for lat/lon information.
- As suggested, the outside borderline box is removed for the figures.

Figure 5

- The Legend can be improve
- Improved the legend
- Please re-check the font size of the caption
- Changed the font size to maintain consistency

Figure 7

- Please put dot line between AEZs. The series overlap can be made to 0%. Do the same for Fig. 9 as well.
- As suggested by the reviewer, dot line is added between AEZs in figure 7 and the series overlap is changed to “0%”

Figure 8

- Series overlap can be made to 0% for each GCM
- Changed as suggested

Table 1

- Table can be improved. No color shading should be use and a proper bordering line should be employ. Please refer to other research article on how to prepare a proper table for research publication. Do the same for all Table.
- Colour shading is removed for the table as suggested.
