# Supplementary material for: Simulating adaptation strategies to offset potential impacts of climate variability and change on maize yields in Embu County, Kenya
Source: PLoS One. 2020 Nov 5;15(11):e0241147. doi: 10.1371/journal.pone.0241147 (PMC7643979; doi:10.1371/journal.pone.0241147)
Supplement: Attachment — Submitted filename: Comments.docx [file pone.0241147.s005.docx]

**General Comments**

The manuscript has been greatly improved by the authors since the first revision. The manuscript section/sub-section has been arranged in a good order. There are some minor revision needed, given in the specific comments below.

**Specific comments**

Abstract:

- Line 20: than rest > than the rest

1. Introduction

- Line 78: projected climate conditions > projected climate conditions on crop growth
- Line 126: maximum and minimum temperatures > Tmax and Tmin

2.2 Current climate variability and future climate conditions

- Line 172: All of the four stations > All stations
- Line 198: Representative Concentration Pathways (RCPs) > RCPs
- Line 200: W m−2 > W m^-2^
- Line 209: Arrange the equation properly
- Line 227: as use > the usage
- Line 234: one GCM, major > one GCM, as major

2.4.1 Farm and farm management data:

- Line 264: Delete the colon (:). Do the same for the rest.

2.4.2 Soil data

- Line 283: form > from
- Table 2: What is soil type for Machanga? Please explain briefly the missing soil parameter in the discussion.

2.4.4 Model calibration and validation

- Line 318: CSMs s were > CSMs were
- Line 318-319: Please rephrase the sentence.
- Line 324: Table 4 > Table 4.
- Line 324: of 160 > from 160
- Line 328: are generally > are found to be generally

2.4.5 Crop simulations

- Line 353: left over > leftover

3.1 Trends in observed climatic conditions

- Line 403: expected have > expected to have

3.2 Projected 406 changes in climate conditions

- Line 413: compared to to other > compared to other
- Line 446: What is this sentence? "Impacts of climate change on maize productivity of the impact on maize." Is this a new sub-heading?

4.1 Climate Variability

- Line 631: log-term > long-term

4.3 Impacts of climate variability and change on maize production

- Line 714: and by when > and when
- Line 712-716: The sentences is too long. It can be separated into two sentences.
