# Supplementary material for: Simulating adaptation strategies to offset potential impacts of climate variability and change on maize yields in Embu County, Kenya
Source: PLoS One. 2020 Nov 5;15(11):e0241147. doi: 10.1371/journal.pone.0241147 (PMC7643979; doi:10.1371/journal.pone.0241147)
Supplement: Attachment — Submitted filename: Comments_10Oct.docx [file pone.0241147.s006.docx]

**Response to comments and suggestions**

To

Shamsuddin Shahid

Academic Editor

PLOS ONE

Dear Editor

We truly appreciate the thorough review and comments offered on our article. After careful consideration of the general and specific comments suggested, we have drafted our responses as shown below and made appropriate changes in the attached revised article

Reviewer #1: (PONE-D-20-14901) Simulating possible Impacts of climate variability and change on Maize production in Embu County, Kenya.

**General Comments**

The manuscript has been greatly improved by the authors since the first revision. The manuscript section/sub-section has been arranged in a good order. There are some minor revision needed, given in the specific comments below.

- As suggested by the reviewer, minor changes are incorporated in the manuscript

**Specific comments**

Abstract:

- Line 20: than rest > than the rest

Changed as suggested

1. Introduction

- Line 78: projected climate conditions > projected climate conditions on crop growth

Changed as suggested

- Line 126: maximum and minimum temperatures > Tmax and Tmin

Changed as suggested

2.2 Current climate variability and future climate conditions

- Line 172: All of the four stations > All stations
- Changed as suggested
- Line 198: Representative Concentration Pathways (RCPs) > RCPs
- Changed as suggested
- Line 200: W m−2 > W m^-2^

Changed as suggested

- Line 209: Arrange the equation properly

Changed as suggested

- Line 227: as use > the usage

Changed as suggested

- Line 234: one GCM, major > one GCM, as major

Changed as suggested

2.4.1 Farm and farm management data:

- Line 264: Delete the colon (:). Do the same for the rest.

Changed as suggested and in rest of the sub-sections, colon (:) is deleted

2.4.2 Soil data

- Line 283: form > from

Changed as suggested

- Table 2: What is soil type for Machanga? Please explain briefly the missing soil parameter in the discussion.

Soil Type for Machanga is “Xanthic Ferralsol” and the missing soil texture details are incorporated in the table as suggested

2.4.4 Model calibration and validation

- Line 318: CSMs s were > CSMs were

Whole sentence is revised

- Line 318-319: Please rephrase the sentence.
- Whole sentence is revised as instructed
- Line 324: Table 4 > Table 4.

Changed as suggested

- Line 324: of 160 > from 160

Changed as suggested

- Line 328: are generally > are found to be generally

Changed as suggested

2.4.5 Crop simulations

- Line 353: left over > leftover

Changed as suggested

3.1 Trends in observed climatic conditions

- Line 403: expected have > expected to have

Changed as suggested

3.2 Projected 406 changes in climate conditions

- Line 413: compared to to other > compared to other

Changed as suggested

- Line 446: What is this sentence? "Impacts of climate change on maize productivity of the impact on maize." Is this a new sub-heading?

Sub-heading in the previous version, this is now deleted from the manuscript

4.1 Climate Variability

- Line 631: log-term > long-term

Changed as suggested

4.3 Impacts of climate variability and change on maize production

- Line 714: and by when > and when

Revised the entire sentence

- Line 712-716: The sentences is too long. It can be separated into two sentences.

As suggested by the reviewer, the whole sentence is revised and separated into two small sentences.
